# Cunnilingus Apparently Increases Duration of Copulation in the Indian Flying Fox, *Pteropus giganteus*

**DOI:** 10.1371/journal.pone.0059743

**Published:** 2013-03-27

**Authors:** Jayabalan Maruthupandian, Ganapathy Marimuthu

**Affiliations:** Department of Animal Behaviour and Physiology, School of Biological Sciences Madurai Kamaraj University, Madurai, India; University of Regina, Canada

## Abstract

We observed a total of 57 incidences of copulation in a colony of the Indian flying fox, *Pteropus giganteus*, over 13 months under natural conditions. The colony consisted of about 420 individuals, roosting in a *Ficus religiosa* tree. Copulations occurred between 07.00 h and 09.30 h from July to January, with more occurring in October and November. Initially males groomed their penis before approaching a nearby female. Females typically moved away and males followed. When the female stopped moving, the male started licking her vagina (cunnilingus). Typically each bout of cunnilingus lasted for about 50 s. In 57 out of 69 observations, the male mounted the female and copulated. The duration of copulation varied from 10 to 20 sec. After completion of copulation, the male continued cunnilingus for 94 to 188 sec. The duration of pre-copulatory cunnilingus and copulation was positively correlated whereas, the duration of pre- and post-copulatory cunnilingus was negatively correlated. Apart from humans, oral sex as foreplay prior to copulation is uncommon in mammals. Another pteropodid bat, *Cynopterus sphinx* exhibits fellatio with females licking the penis of males during copulation. It appears that bats, especially pteropodids perform oral sex, either cunnilingus or fellatio, possibly for achieving longer copulation.

## Introduction

Observing copulatory activity of bats under natural conditions is difficult mainly because they live in inaccessible sites such as caves, crevices and tree holes. Among the 1100 species of bats, copulatory activity has been described for less than ten including the hammer-headed bat *Hypsignathus monstrosus* [1], the Indian flying fox *Pteropus giganteus* [2], [3], the Australian flying fox *P. poliocephalus* [4], the little brown bat *Myotis lucifugus* [5], the Brazilian free-tailed bat *Tadarida brasiliensis* [6], the common vampire bat *Desmodus rotundus* [7], and the short-nosed fruit bat *Cynopterus sphinx* [8]. Observations of social organization [9] including behavioural repertoire and copulation [2], [3] of *P. giganteus* is less difficult, mainly because the majority of individuals roost in trees. Our preliminary observations showed that during the breeding season, *P. giganteus* males follow females during courtship while females move away. Similarly, female black flying foxes *P. alecto* [10] move away when males approach them. When the females stop moving, the males begin to sniff and lick their uro-genital regions. Female C. *sphinx* licks the penis of males during copulation (fellatio) [8]. These observations on *P. alecto* and *C. sphinx* led us to assess whether individuals of *P. giganteus* exhibit any such behaviour associated with copulation. Our subsequent observations revealed that males exhibit cunnilingus (licking vagina of females). We describe these behaviours and their influence on copulation.

## Materials and Methods

We conducted the study from July 2010 to July 2011 near the village of Nallachampatti (9°58′N, 77°47′E), approximately 32 km west of Madurai Kamaraj University campus. A colony of about 420 *P. giganteus* occupied a single *Ficus religiosa* tree (Family: Moraceae), situated adjacent to private agricultural land. We recorded the timings of emergence and returning flights, behaviours observable during daytime such as copulation together with the influence of ambient temperature, humidity and rain. These observations were made twice a month by using binoculars (Balileo 20×50 gross feld) from 0230 h (day 1) to 2330 h (day 2) for a total of 1170 hours. Copulation occurred during the morning (0700–0930 h) between July and January. In addition to visual observations, we made videographs (Sony handycam DCR-SR47) and photographs (Nikon D3000 with 18–200 mm lens) of the bats from which to prepare illustrations and supporting documents. Humidity and temperature in the study area were 68.5±2.2% and 28.7±0.7°C (n = 26 for both), respectively. Because movement of people was common in the area, we presumed that our presence did not disturb the bats.

### Statistical Analysis

We used Pearson correlation and linear regression analyses to assess whether the duration of cunnilingus before copulation and the duration of copulation were associated. Similarly, we evaluated whether there was an association between the duration of pre- and post-copulatory cunnilingus. Values are given as means plus or minus one standard error. All tests employed an alpha value of 0.05.

### Ethics Statement

We neither captured bats nor disturbed the individuals of the colony of *P. giganteus*. The entire study relied on visual observations. Although ethical approval was not mandatory, our protocol was approved by the “Internal Research Review Board (IRB), Ethical Clearance (EC), Biosafety and Animal Welfare Committee” of the Madurai Kamaraj University.

## Results and Discussion

**Figure 1:**
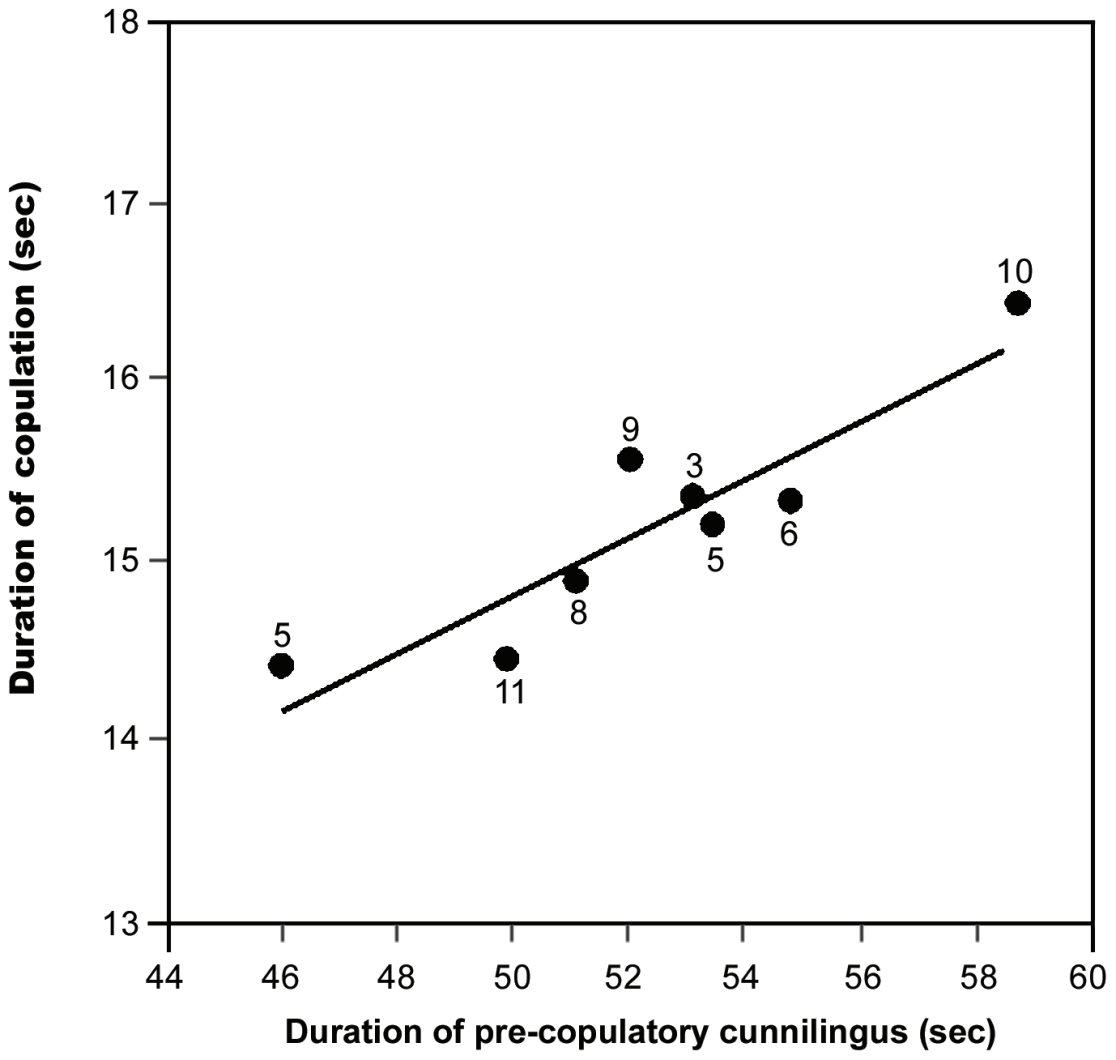
Relationship between duration of pre-copulatory cunnilingus and copulation. Circles and numbers indicate average duration of copulation and *n* value, respectively.

**Figure 2:**
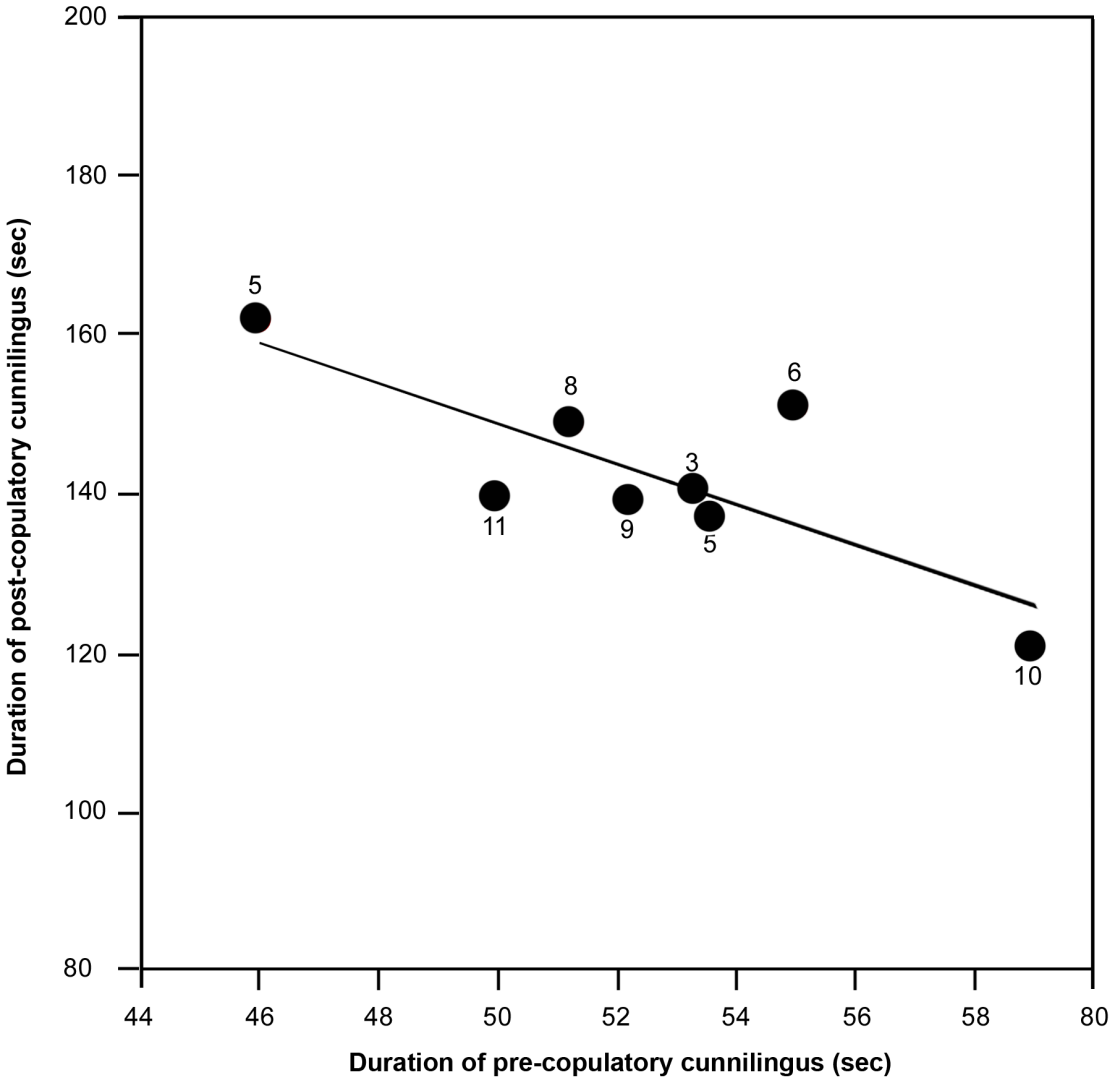
Relationship between duration of pre- and post-copulatory cunnilingus. Circles and numbers indicate average duration of cunnilingus and *n* value, respectively.

We observed a total of 57 copulations, all between 07.00 h and 09.30 h from July 2010 to July 2011. More copulations occurred during October and November. Initially a male groomed its genital organ, which led to erection of the penis. Afterwards, the male moved towards a nearby female with gentle wing flapping and touched with its wing (Video S1). In 57 out of 69 observations, the female moved away and the male followed. After 12–75 s, the female stopped moving and the male craned its neck to reach the female’s vagina and started licking it (Video S1). The female was alert and let the male exhibit cunnilingus for 50.5±2.12 s (n = 57). The male then mounted the female (Video S1). The act of copulation lasted 14.9±0.38 s, (n = 57). Afterwards, the male dismounted and resumed cunnilingus (Video S1) in all the observations (n = 57). The female did not show any movement during this post-copulatory cunnilingus (Video S1), which was always longer (146.4±4.0 s; n = 57) than that before copulation occurred. Although prolonged rituals and competition between males occurred in the remaining 12 observations, we could not follow them as it was beyond the capacity of our visual observation. We found a positive relationship between the duration of pre-copulatory cunnilingus and copulation (Pearson correlation: r_6_ = 0.91, P<0.05; Fig. 1). Thus the duration of pre-copulatory cunnilingus was related to increase in the duration of copulation. Whereas, we found a negative relationship between the duration of pre- and post-copulatory cunnilingus (Pearson correlation: r_6_ = 0.79, P<0.05; Fig. 2).

Our observations suggest that cunnilingus is an important prerequisite for male *P. giganteus* making the female receptive for copulation. The positive correlation between the duration of pre-copulatory cunnilingus and copulation is consistent with this suggestion. Pre-copulatory cunnilingus may enhance the arousal and lubrication of the female as suggested for fellatio in *C. sphinx* [8]. Thus, the longer the duration of cunnilingus the greater the duration of copulation. Markus [10] reported that male *P. alecto* also exhibits courtship display by sniffing and licking the female’s genital organ before copulation, but she did not quantify the duration of copulation. At the completion of the initial copulation, unlike *P. alecto* [10], *P. giganteus* males never attempted to resume copulation. Male *P. alecto* never repeated vaginal licking after the initial copulation. Instead, they began to groom or lick their own penis [10]. We never observed *P. giganteus* grooming its penis after copulation. The longer duration of post-copulatory cunnilingus in *P. giganteus* compared to that observed before copulation in all cases may be due mainly to the fact that females made no movements after copulation.

A possible functional explanation for the association of cunnilingus with copulation may be sperm competition. Before copulation, *P. giganteus* males lick females’ genitals to remove sperm from males that might have mated with her earlier, as occurs in ‘cloaca pecking’ by dunnocks, *Prunella modularis* [11]. However, whether several *P. giganteus* males mate with the same female over a short time period is not known. The positive relationship between the duration of pre-copulatory cunnilingus and copulation supports the sperm competition hypothesis (lick for longer and copulate for longer increases the chances of paternity). In this context, cunnilingus would be maladaptive after mating as there is a risk of removing the male’s own sperm. Observation at close-range is needed to find out whether the male’s tongue enters the vagina or not. Nevertheless, the negative relationship between the duration of pre- and post-copulatory cunnilingus suggests the possibility of sperm competition. Thus, the male removes the sperm of competitors but apparently not its own – lick for longer before but less after copulation, if paternity uncertainty is high. Hosken [12] suggested that the risk of sperm competition among pteropodids increases with group size, testes mass and conspecific proximity. Further study on the association between cunnilingus and copulation with reference to group size and testes mass will likely lead to greater understanding of sperm competition in *P. giganteus*.

Ours is the first evidence for cunnilingus occurring both before and after copulation in bats under natural conditions. Oral sex was observed in *C. sphinx* under captive conditions, in which females lick the male’s penis during dorsoventral copulation [8]. Fellatio in *C. sphinx* occurs simultaneously with copulation, whereas cunnilingus takes place in *P. giganteus* both before and after copulation. It appears that male *P. giganteus* and *P. alecto* [10] and female *C. sphinx* [8] employ oral sex as a pre-requisite for successful copulation. Similar studies should be carried out on other bat species, especially pteropodids, to establish whether oral sex, in association with copulation, is common in this group.

## Supporting Information

Video S1 **Sequence of male** ***P. giganteus*** **approaching female, the male exhibiting pre-copulatory cunnilingus, the male mounting the female, dorso-ventral copulation and the male exhibiting post-copulatory cunnilingus.**
(WMV)
